# Sources of Health Information and Their Impacts on Medical Knowledge Perception Among the Saudi Arabian Population: Cross-Sectional Study

**DOI:** 10.2196/14414

**Published:** 2020-03-19

**Authors:** Shatha A Alduraywish, Lamees A Altamimi, Rawan A Aldhuwayhi, Lama R AlZamil, Luluh Y Alzeghayer, Futoon S Alsaleh, Fahad M Aldakheel, Shabana Tharkar

**Affiliations:** 1 Department of Family and Community Medicine College of Medicine King Saud University Riyadh Saudi Arabia; 2 College of Medicine King Saud University Riyadh Saudi Arabia; 3 Department of Clinical Laboratory Sciences College of Applied Medical Sciences King Saud University Riyadh Saudi Arabia; 4 Prince Sattam Chair for Epidemiology and Public Health Research College of Medicine King Saud University Riyadh Saudi Arabia

**Keywords:** health information sources, health perception, medical information sources, satisfaction, social media, trust

## Abstract

**Background:**

Having a reliable source for health information is vital to build a strong foundation of knowledge, especially with the current revolution of the internet and social media, which raises many concerns regarding harmful effects on the health of the public. However, there are no studies on how the Saudi Arabian population seeks health information. Details about the most used and trusted sources of health information among the public will help health authorities and public awareness accounts on social media to effectively disseminate health information.

**Objective:**

To investigate the types of sources accessed by the Saudi Arabian population while seeking health information, as well as their level of trust in the sources and to assess the impact of these sources on their perception of medical knowledge and health decision-making.

**Methods:**

A cross-sectional study was conducted to meet the objectives. The study population included both men and women who were aged 16 years or more and visited primary care clinics at King Khalid University Hospital. Four hundred and thirteen participants were sampled using the simple random method, and a self-administered questionnaire was used to collect data. The data were analyzed using SPSS software (IBM Corp, Armonk, New York, USA).

**Results:**

A total of 413 participants were included in this study, and of these, 99 (24.0%) were males and 206 (49.9%) had a bachelor’s degree. Doctors were chosen as the first source of information by 87.6% (283/323) of the participants, and they were completely trusted by most of the population (326/411, 79.3%). The second most commonly used source was pharmacists (112/194, 57.7%), and they were partially trusted by 41.4% (159/384) of the participants. Internet searches, social media, and traditional medicine were not prioritized by most of the participants as the first or second source of health information. The majority of the participants did not trust information obtained from social media, and WhatsApp was the most untrusted source. Almost half of the respondents (197/413, 47.7%) acknowledged that various sources of information can often help them understand their health problems. However, the majority disagreed on substituting a doctor’s prescription with information obtained from the internet or a friend or relative.

**Conclusions:**

Although physicians were preferred and highly trusted, internet sources appeared to impact the medical knowledge of the population. The population still preferred to use internet search to obtain health information prior to a doctor’s visit.

## Introduction

The current sources of health information are diverse, and they influence the perception of medical knowledge among the Saudi Arabian population. Having a reliable source of health information is critical for building a strong foundation of knowledge about health among the public, especially with the current revolution of the internet and social media. According to the latest statistics, more than 50% of the global population uses the internet. Moreover, more than 64% of the Saudi Arabian population uses the internet, and this number has been increasing every year [1]. Although social media, websites, and internet search engines are considered as easily accessible sources of medical information, these sources still contain ambiguities. There is increasing evidence that the instant exchange of news from random resources and the lack of verification and determination of the accuracy and credibility of the information being shared by nonprofessionals raise many concerns about the harmful effects on a person’s health [2].

Determining the frequency and characteristics of people who are likely to use the internet and social media to obtain their medical knowledge may guide both clinicians and the public. In addition, it is important from a public health perspective to appreciate the probable subsequent health outcomes. Moreover, the health information sought from different sources and trusted by the public could tremendously influence the quality of health care rendered, as it could affect their judgment of a physician’s medical opinion and, in turn, affect their health decision-making. Previous studies have found that the commonly used sources of health information are the internet [1,3,4], physicians [5], social media [6], radio and television [7], pharmacists [8], and parents [9]. It has been reported that more than half of the public widely use the internet to seek health information [1,10,11], and the majority are young adults [1]. In a study conducted in Qatar, it was found that about 37.8% of Qataris seek health information from family and friends and 31.2% rely on primary health care centers as a source of health information [1].

In Australia, the use of the internet as a source of health information by the university population (students and staff) is greater than the use by the low or middle socioeconomic population [12]. It has been reported that gender, age, and educational level play significant roles in predicting the source of medical information. However, irrespective of the source of consultation, women are generally more interested in seeking health information than men, and the majority of those who seek medical information from the internet are women [1]. In comparison with the different available sources of health information, health care providers have been reported to be the most trusted source among different adult populations in the United States [11]. The majority of those who trust health care providers as the source of their health information are female, young, and educated [11], and this is irrespective of their socioeconomic status [12].

The roles of family and friends, pharmacists, and local alternative medicine practitioners are rarely discussed. As such, additional research is needed to evaluate the use of the internet and social media as tools for medical decision-making. Familiarity with the most used and trusted sources of health information among the public will assist health authorities and public awareness accounts on the internet and social media in accurately and carefully disseminating health information among the public, who should be educated and advised about the most credible sources of health information.

It is important to mention that social media platforms in Saudi Arabia, specifically WhatsApp, are not only used as communication tools among family and friends. The easy broadcasting characteristics of WhatsApp allow the population to exchange numerous videos, news, and messages. As these broadcasts continue to circulate among the public, the source of the information can no longer be tracked and verified. Health information is one of the most commonly exchanged messages and are most frequently completely false. This is why we express our concerns in this paper, and we investigated the level of trust of information broadcasted through WhatsApp and assessed whether it affects a patient’s health-seeking behavior and medical decisions.

To our knowledge, there have been no studies on how the Saudi Arabian population seeks information regarding their health. Therefore, the purposes of this study were to (1) investigate the different sources that the Saudi Arabian population uses and trusts for medical information and (2) assess the impact of these sources on medical knowledge and the patient’s health decision-making. We hypothesized that more than half of the Saudi Arabian population uses the internet and social media to obtain medical information.

## Methods

### Study Setting and Population

We conducted an observational cross-sectional study to address our research objectives. The study was conducted in primary care clinics at King Khalid University Hospital (KKUH), which is a large 1000-bed tertiary-care hospital in the northern part of Riyadh, Saudi Arabia. KKUH provides primary, secondary, and tertiary care services to a large patient-catchment area and government-funded free preventive and curative services. A random selection was performed of all attending Saudi male and female patients aged 16 years or more, which is the cutoff age for patients attending the adult primary care clinics. There were no exclusion criteria other than age and nationality.

The required ethical approval was obtained from the institutional review board of King Saud University. Informed consent was obtained from the participants, and the confidentiality of the information and privacy of the participants were protected throughout the study.

### Sample Size Estimation

According to a recent study from the neighboring gulf region, 71% of the Qatari population uses the internet to seek health-related information. With a 95% CI and precision of 5, the sample size in this study was estimated at 386, which was further increased to 425 considering a nonresponse rate of 10%.

### Recruitment of Participants

Four hundred and thirteen participants were recruited using simple random sampling, which was conducted through a random number generator software.

### Data Collection Tools

The data were collected using a self-administered questionnaire or an interview (in the case of illiterate participants). The questionnaire had three main parts. The first part assessed the demographic data of the participants, such as age, gender, area of residence, educational level, and current occupation. The second part assessed the different used and trusted sources of medical information. It contained two questions. The first question concerned the ranking of the most used source, and the second question concerned the extent of trust in the sources chosen in the first question. The third part of the questionnaire was designed to explore the impacts of those sources on the participant’s medical knowledge and their effects on health decision-making.

### Tool Validation

The development of the questionnaire survey was based on a literature review, and some of the questions in the second part of the questionnaire were adapted from the Health Information National Trends Survey (HINTS). We used the information sources mentioned in the HINTS [13]. For the third part, we adopted most of the questions from the behavioral involvement subscale of the Assessment of Preferences for Self-Treatment and Information in Health Care survey [14]. A pilot study was conducted to assess the time needed to complete the questionnaire and the understandability of the included questions.

### Statistical Analysis

The data were analyzed using IBM SPSS Statistics for Windows version 23.0 (IBM Corp, Armonk, New York, USA). The data are expressed using frequencies and percentages for categorical variables and means and SDs for continuous variables. The chi-square test was used for categorical variables. The association between the sociodemographic characteristics and the sources of health information was examined using logistic regression, and the results are expressed with ORs and 95% CIs. A two-sided *P* value of <0.05 was considered statistically significant.

## Results

### Characteristics of the Study Participants

Of 425 distributed questionnaires, 413 completed questionnaires were obtained (response rate of 88%). Twenty participants were illiterate and thus were interviewed. The majority of the respondents were female (314/413, 76.0%). The study sample was well distributed across all age groups, and most of the population was literate. The overall health status of the participants was good. However, 130 (31.6%) had chronic diseases, and 52 (40.0%) had diabetes, 44 (33.8%) had hypertension, and 34 (26.2%) had asthma. The demographic data are summarized in Table 1.

**Table 1:** Characteristics of the study participants.

Characteristic		Value, n (%)
**Gender**		
	Male	99 (24.0)
**Age (years)**		
	16-25	98 (24.0)
	26-35	138 (33.7)
	36-45	91 (22.2)
	46-55	64 (15.6)
	≥56	18 (4.4)
**Residency (province)**		
	Central	362 (87.7)
	Northern	27 (6.5)
	Southern	9 (2.2)
	Eastern	6 (1.5)
	Western	9 (2.2)
**Educational level**		
	Elementary school	16 (3.9)
	Intermediate school	22 (5.3)
	High school	86 (20.8)
	Diploma	32 (7.7)
	Bachelor studies	206 (49.9)
	Postgraduate studies	31 (7.5)
	Illiterate	20 (4.8)
**Occupation**		
	Student	64 (5.6)
	Government employee	115 (28.0)
	Private sector employee	55 (13.4)
	Retired	21 (5.1)
	No occupation	155 (37.8)
**Field of occupation**		
	Education	125 (45.6)
	Medical	29 (10.6)
	Military	9 (3.3)
	Business	20 (3.7)
	Others	91 (33.2)
**Self-assessment of health status**		
	Very weak	2 (0.5)
	Weak	18 (4.4)
	Good	253 (61.3)
	Excellent	140 (33.9)
Reported history of chronic disease		130 (31.6)

### Different Sources and Choice of Preference for Health Information

Doctors were the most favored choice for the majority of the study population (283/323, 87.6%); however, a smaller fraction rated them as second (18/323, 5.6%), third (12/323, 3.7%), and fourth preferences (10/323, 3.1%). Pharmacists were rated as the second most favored choice after doctors by a little more than half of the population (112/194, 57.7%). Social media was least preferred as the first choice (2/105, 1.9%). Even doctors who are on social media were less preferred as the first choice (19/157, 12.1%) (Table 2).

**Table 2:** Different sources used for health information.

Source of information	Respondents, n or n (%)		Ranking, n (%)									
			First		Second		Third			Fourth or more		
	Total	Female	Total	Female	Total	Female		Total	Female		Total	Female
Doctor	323	240 (74.3)	283 (87.6)	211 (87.9)	18 (5.6)	14 (5.8)		12 (3.7)	9 (3.8)		10 (3.1)	6 (2.5)
Pharmacist	194	137 (70.6)	13 (6.7)	12 (8.8)	112 (57.7)	74 (54.0)		31 (16.0)	24 (17.5)		38 (19.6)	27 (19.7)
Traditional medicine practitioner	78	50 (64.1)	5 (6.4)	2 (4.0)	6 (7.7)	5 (10.0)		15 (19.2)	9 (18.0)		52 (66.7)	34 (68.0)
Social media	105	69 (65.7)	2 (1.9)	2 (2.9)	11 (10.5)	7 (10.1)		17 (16.2)	10 (14.5)		75 (71.4)	50 (72.5)
Doctors who are on social media	157	122 (77.7)	19 (12.1)	16 (13.1)	48 (30.6)	38 (31.1)		36 (22.9)	29 (23.8)		54 (34.3)	39 (32.0)
Family and friends	187	133 (71.1)	14 (7.5)	9 (6.8)	26 (13.9)	21 (15.8)		46 (24.6)	34 (25.6)		101 (54.0)	69 (51.9)
Internet search	184	132 (71.7)	28 (15.2)	20 (15.2)	41 (22.3)	31 (23.5)		45 (24.5)	31 (23.5)		70 (38.0)	50 (37.9)
Articles	95	64 (67.3)	2 (2.1)	1 (1.6)	7 (7.4)	4 (6.3)		15 (15.8)	11 (17.2)		71 (74.7)	48 (75.0)
Television and radio	122	81 (66.3)	5 (4.1)	2 (2.5)	14 (11.5)	9 (11.1)		19 (15.6)	12 (14.8)		84 (68.9)	58 (71.6)
Courses and campaigns	93	66 (70.9)	13 (14.0)	11 (16.7)	19 (20.4)	15 (22.7)		61 (65.6)	40 (60.6)		—^a^	—^a^

^a^None of the participants selected this source.

### Level of Trust in Each Source of Health Information

The levels of trust in the sources of information are presented in Table 3. Doctors were the most trusted, and there was either complete trust (326/411, 79.3%) or partial trust (85/411, 20.6%), and no participant reported distrusting them. Pharmacists and traditional practitioners were partially trusted, with similar ratings; however, they were also distrusted by some participants. The majority of the participants did not trust the information obtained from social media. Gender differences were observed in the level of trust in social media, wherein more women showed distrust in social media than did men (*P*=.01) (Table 3).

In addition, the population distributions for the first choice of the source of health information and complete trust in the first choice are presented in Table 4.

**Table 3:** The level of trust in each source of health information.

Source of information	Number of respondents, n or n (%)		Level of trust, n (%)							
			Completely trusted			Partially trusted			Not trusted	
	Total	Female	Total	Female	Total		Female	Total		Female
Doctor	411	312 (75.9)	326 (79.3)	244 (74.8)	85 (20.6)		68 (80.0)	—^a^		—^a^
Pharmacist	384	287 (74.7)	23 (6.0)	1 (4.3)	159 (41.4)		89 (56.0)	202 (52.6)		197 (97.5)
Traditional medicine	271	209 (66.6)	9 (3.3)	5 (55.6)	115 (42.4)		85 (74.0)	147 (54.2)		119 (81.0)
WhatsApp	331	233 (70.4)	22 (6.6)	3 (13.6)	123 (37.1)		69 (56.1)	186 (56.1)		161 (86.6)
Snapchat	318	219 (68.9)	22 (6.9)	6 (27.3)	127 (39.9)		83 (65.4)	169 (53.1)		130 (77.0)
Twitter	273	205 (74.8)	5 (1.8)	3 (60.0)	139 (50.9)		104 (74.8)	130 (47.6)		98 (75.4)
Family and friends	360	271 (75.3)	27 (7.5)	23 (85.2)	265 (73.6)		199 (75.1)	68 (18.9)		49 (72.1)
Internet search	353	266 (75.4)	28 (7.9)	20 (71.4)	249 (70.5)		187 (75.1)	76 (21.5)		59 (77.6)
Television and radio	314	234 (74.5)	33 (10.5)	22 (66.6)	197 (62.7)		147 (74.6)	84 (26.8)		65 (77.4)

^a^None of the participants selected this option.

**Table 4:** Distributions of the population for the first choice and complete trust in the information.

		First choice, n (%)			Complete trust, n (%)			
		Medical^a^	Media^b^	Others^c^		Medical	Media	Others
**Age (years)**								
	16-25	73 (83.0)	6 (7.0)	8 (9.0)		77 (93.0)	0 (0.0)	5 (6.0)
	26-45	151 (75.0)	38 (19.0)	12 (6.0)		179 (97.0)	3 (2.0)	3 (2.0)
	≥46	67 (92.0)	3 (4.0)	3 (4.0)		73 (95.0)	1 (1.0)	3 (4.0)
**Gender**								
	Male	72 (78.0)	10 (11.0)	10 (11.0)		82 (99.0)	0 (0.0)	1 (1.0)
	Female	221 (82.0)	37 (14.0)	13 (5.0)		250 (95.0)	4 (2.0)	10 (4.0)
**Chronic diseases**								
	Present	96 (83.0)	18 (16.0)	1 (1.0)		110 (94.0)	0 (0.0)	7 (6.0)
	Absent	196 (79.0)	29 (12.0)	22 (9.0)		222 (97.0)	4 (2.0)	4 (2.0)
**Occupation**								
	Employed	49 (86.0)	3 (5.0)	5 (9.0)		47 (96.0)	0 (0.0)	2 (4.0)
	Retired	115 (77.0)	23 (15.0)	12 (8.0)		136 (96.0)	3 (2.0)	3 (2.0)
	Unemployed	17 (89.0)	1 (5.0)	1 (5.0)		19 (96.0)	0 (0.0)	1 (5.0)
	Student	111 (82.0)	20 (15.0)	5 (4.0)		128 (96.0)	1 (1.0)	4 (4.0)
**Education**								
	School	92 (84.0)	11 (10.0)	6 (6.0)		101 (97.0)	1 (1.0)	2 (2.0)
	Higher education	185 (78.0)	35 (15.0)	16 (7.0)		215 (96.0)	3 (1.0)	7 (3.0)
	Illiterate	17 (89.0)	1 (5.0)	1 (5.0)		17 (89.0)	0 (0.0)	2 (11.0)

^a^Medical includes doctors, pharmacists, and traditional medicine practitioners.

^b^Media includes WhatsApp, Twitter, Snapchat, and the internet.

^c^Others include family, friends, courses, campaigns, television, and radio.

### Impacts of the Sources of Health Information on the Individual’s Health Perception and Clinical Decision-Making

**Figure 1:**
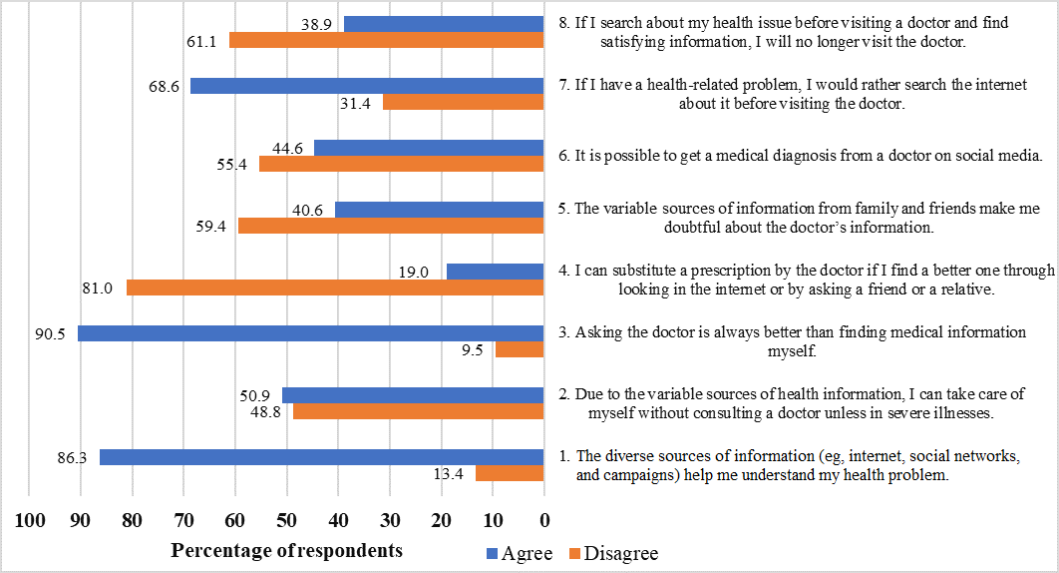
Impacts of sources of health information on an individual’s health perception and clinical decision-making.

Although around 86.0% (355/413) of the population reported using diverse sources for health information, 90.0% (371/413) preferred to seek help from doctors. Additionally, 68.0% (280/413) perceived to seek information from other sources only prior to a doctor’s visit. The majority disagreed on substituting a doctor’s prescription with information obtained from the internet or a friend or relative (Figure 1).

## Discussion

### Principal Findings

This study aimed to investigate the different sources that the Saudi Arabian population uses and trusts for their health information and to assess the impact of these sources on an individual’s medical knowledge and health decision-making. The findings from this study revealed that doctors were the most commonly used source of health information, followed by internet search, whereas courses and campaigns were the least common sources. Doctors were the most trusted source, and unexpectedly, social media was the least trusted source. Although about half of the population admitted that various sources helped them understand their health problems, they prioritized doctor’s advice over other sources.

In this study, among the sources of information, we found that less than half of the Saudi Arabian population used the internet as a source of information. Similarly, more than half of the population did not use social media as one of the principal sources for health information. These findings did not support our study hypothesis. However, we found that more than half of the population (283/323, 87.6%) considered doctors as a primary source of information. Moreover, doctors were completely trusted for medical information by more than half of the population (326/411, 79.3%), which is similar to the findings in the study by Hesse et al, in which health care providers were the most trusted source among different populations [11].

However, 20.6% (85/411) of the population partially trusted their health care providers. This raises many concerns regarding the quality of medical care provided, as there might be an issue with the doctor-patient relationship. Many reasons could contribute to this diminished level of trust. For example, problems in communication between a doctor and patient could result in misunderstanding of the patient’s health issues, which could lead to patient dissatisfaction with the consultation. As a result, complete information will not be disclosed if the patient does not trust the doctor [15]. This affects the efficiency in patient management and consequently the trust in the doctor’s medical judgment.

Furthermore, more than half of the population stated that they will not substitute a doctor’s prescription with the information obtained from other sources. This finding is similar to that in the study by Diaz et al [10], confirming that other sources of information do not affect the certainty of the doctor’s information. In addition, it supports that initially seeking a doctor’s advice is prioritized over searching for other sources of information.

Pharmacists were considered as the second most commonly used source of information (112/194, 57.7%), although they were partially trusted by 41.4% (159/384) of the population. However, only a few respondents considered pharmacists as the first source of information (13/194, 6.7%). The fact that pharmacists follow doctors as a source of medical information is a good indicator of the population’s perception regarding the priorities for obtaining information from health care practitioners.

Despite the common use of traditional medicine by the Saudi Arabian population [16], our data revealed that traditional medicine practitioners were not trusted by the majority of the Saudi Arabian population. As a result, most of the population did not consider traditional medicine practitioners as a preferred source of information. These findings indicate that although individuals believe in traditional medicine, they do not obtain this kind of treatment from its practitioners. The reason for this is that a lot of traditional medicine practitioners in Saudi Arabia are not certified. Instead, most of them are owners of small shops of herbal remedies and practice this field of medicine as a hobby, and they lack a qualification and scientific background in this field.

Social media was not the most used or trusted source of health information by the majority of the participants. However, in the United States, more than half of the population uses social media to obtain health information [17]. Furthermore, we found that among the different social media applications, WhatsApp was the most untrusted source. These findings seem to be a good indicator of the population’s awareness that unreliable sources should not be used to obtain health information. Similarly, a large percentage of the population did not rely on doctors who are on social media to obtain diagnoses of their conditions, and instead, they used this source to acquire general health information. This finding reflects the population’s awareness of the dangers of a social media diagnosis without a medical consultation, as social media diagnosis can involve many inaccuracies despite the good intentions of the doctors. It emphasizes that doctors who are on social media need to deliver accurate and up-to-date material to avoid potentially harmful effects among their audience.

The majority of the participants did not prioritize internet searching, and more than half partially trusted this source. It is known that internet searches for medical information have well-recognized drawbacks regarding the quality and accuracy of information, as mentioned in the study by Benigeri and Pluye [18]. Surprisingly, courses and campaigns were the least used sources of information, and they were not used at all as the first source of information by the study participants. This reflects a weakness in the role of community awareness of health problems.

### Strengths and Limitations

To our knowledge, this is the first study to discuss the use and trust of the Saudi Arabian population regarding different sources of medical information. The strength of this study is that the sample size was relatively large. However, a few limitations exist. The study population included many female individuals (314/413, 76%) and individuals living in the central province (362/413, 87.7%), which may not be a good reflection of the whole Saudi Arabian population. More diverse patient groups with a larger sample size may be needed to generalize the results. In addition, the data were self-reported, which might involve recall bias. Lastly, we did not investigate the types of information searched for in every source, such as sensitive topics, serious conditions, and educational information, which may change the information source preference.

### Conclusion and Recommendations

This study is the first in Saudi Arabia to investigate the different sources of medical information that are used and trusted by the Saudi Arabian population. We found that doctors were the most used and trusted source, courses and campaigns were the least used sources, and social media, specifically WhatsApp, was the least trusted source. This draws attention to the need to develop well-structured courses and campaigns that meet the needs of the population in an easily understandable way. It also sheds light on the requirement to increase the quality of information provided in the nonmedical field. In addition, further research is needed to understand why a large number of participants only partially trusted their doctors.

## Abbreviations

HINTS: Health Information National Trends Survey
KKUH: King Khalid University Hospital
